# An in vivo imaging approach for simultaneously assessing tumor response and cytotoxicity-induced tissue response in chemotherapy

**DOI:** 10.1007/s10495-025-02118-9

**Published:** 2025-04-26

**Authors:** Steven E. Johnson, Chad R. Haney, Alisha N. Spann, Nigar Khurram, Farres Obeidin, Jungwha Lee, Ming Zhao

**Affiliations:** 1 Department of Medicine, Northwestern University, Chicago, USA; 2 Chemistry of Life Processes, Northwestern University, Evanston, USA; 3 Department of Pathology, Northwestern University, Chicago, USA; 4 Preventive Medicine, Northwestern University, Chicago, USA; 5 Northwestern University, 320 E. Superior St., Searle 10-519, Chicago, IL 60611 USA

**Keywords:** Cytotoxicity, Apoptosis, ^99m^Tc-duramycin, Antitumor efficacy, Adverse effects

## Abstract

In chemotherapeutic treatments, while cancer cells are the primary target, cytotoxic side effects are an important consideration. In the current study, we applied an in vivo imaging tool for characterizing chemotherapeutic response in a preclinical setting. The study focused on simultaneously examining the tumor and tissue response as a result of treatment with bortezomib, a mainstay proteasome inhibitor for treating multiple myeloma, in a preclinical model. OPM-2 tumor-bearing SCID-beige mice were designated as control or treated with bortezomib (1 mg/kg, i.v., every 4 days) (n = 8 per group). ^99m^Tc-duramycin SPECT/CT whole-body scans were acquired 2 days before treatment as baseline and at days 1, 3 and 5 after treatment. Radioactivity uptake in tissues and organs was determined and quantitatively compared between control and bortezomib-treated group at each of the time points. Based on the imaging data, separate groups of tumor-bearing mice (n = 3 each) were included as control and bortezomib treated and the tissues were collected on day 5 for histopathology. In vivo imaging data identified significantly elevated ^99m^Tc-duramycin uptake in the tumor, particularly in tumoral periphery. This was accompanied with signal changes in multiple organs and tissues including the adipose tissue, major bones, abdominal regions, spleen and testes. The imaging findings were consistent with known cytotoxic side effects of bortezomib and were supported by histopathology. The outcome of the study demonstrated potential utilities of the technology by enabling timely determination of the efficacy of anticancer treatments and the effect on collateral tissues as a result of systemic cytotoxic treatment.

## Introduction

Cytotoxic anticancer drugs target tumor cells but also adversely affect susceptible off-target tissues [1–8]. The ability to simultaneously assess antitumor efficacy and collateral tissue damage in a systemic and timely fashion provides an opportunity to obtain critical information for the characterization of new drug candidates and therapies. Such capabilities can ultimately serve to benefit cancer treatment and help minimize side effects for patients.

Cell death, including apoptosis, is an important manifestation of terminal cellular response to cytotoxic stimuli [9–14]. Our prior study has demonstrated that elevated cell death is detectable in vivo and can provide a marker for susceptibility to drug-induced cytotoxic effects [15]. This approach can be applicable without prior knowledge of drug effects, thus serving as a survey of tissue susceptibility in response to drug toxicity. For this purpose, an imaging technique which targets phosphatidylethanolamine (PE) was examined for its capacity in detecting cell death as a surrogate marker for tissue damage. PE is a type of aminophospholipid which is sequestered inside normal cells but is externalized and becomes accessible when a cell is dead or dying [16]. This near-universal marker, which relies on phospholipid redistribution, allows for the detection of multiple forms of cell death regardless of causes or pathways. This is particularly important because there is extensive signaling cross talk among mechanisms of cell death in which different cell/tissue types respond differently to cytotoxic stimuli, wherein the propensity toward one mode of cell death over another may differ [17–19]. The imaging agent, ^99m^Tc-duramycin, binds PE with relatively high affinity and specificity, and with favorable pharmacokinetics [20, 21]. In preclinical settings, small animal single photon emission computed tomography (SPECT) has decent sensitivity and sub-millimeter spatial resolution, which helps overcome partial-volume effect in post-processing.

The goal of the current investigation was to examine the ability to assess tumor and systemic tissue response with whole-body imaging in a minimally invasive and global fashion. Using the whole-body imaging approach, we studied, in a preclinical setting, the tumor and tissue susceptibility to bortezomib, a proteasome inhibitor for treating multiple myeloma, which remains an incurable cancer. The development of proteasome inhibitors showed promise in treating multiple myeloma, and there has been considerable progress in combating this malignancy [22, 23]. However, the benefit of bortezomib can be hindered by poor tolerance due to efficacy-limiting side effects. Main adverse effects from bortezomib treatment include peripheral neuropathy, gastrointestinal disorders, and hematologic issues [24–27]. In light of the challenges and uncertainties facing the development of anticancer drugs and therapies, we tested the hypothesis that a whole-body survey provides useful in vivo indices on the antitumor efficacy and systemic tissue susceptibility. Such information is expected to shed light on the anticancer actions and adverse effects of chemotherapeutic agents, and can help serve to expedite the translation process.

## Results

### Assessment of tumor response

**Fig. 1:**
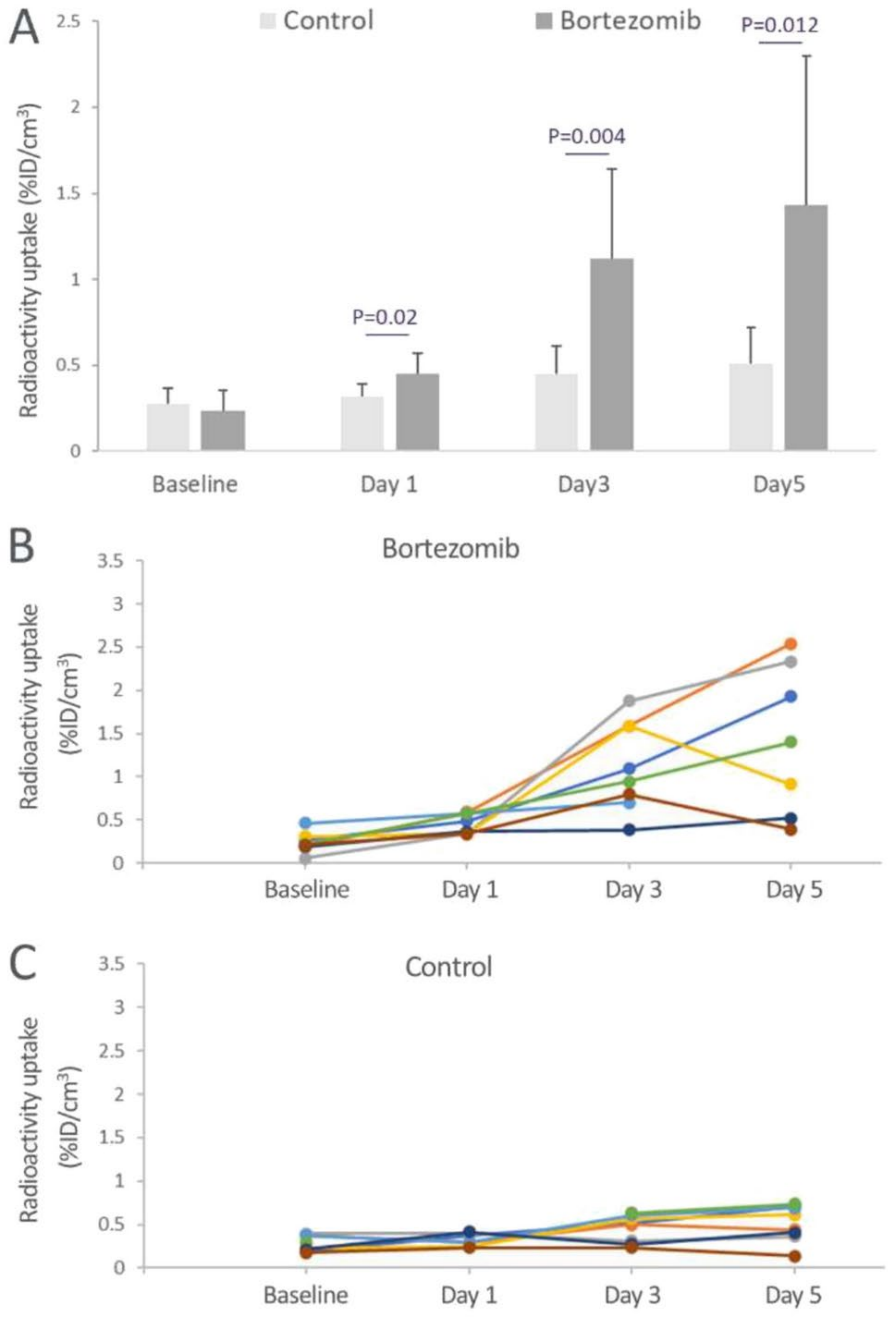
Tumor response with bortezomib treatment. **A** In vivo radioactivity uptake in the tumor between control and bortezomib treated mice before (baseline) and at days 1, 3 and 5 after treatment. Significantly elevated ^99m^Tc-duramycin uptake was identified on day 1 (*P* = 0.02), day 3 (*P* = 0.004) and day 5 (*P* = 0.012) after treatment. **B** and **C** Tumor radioactivity uptake in mice treated with bortezomib or control, respectively. Each individual animal is color-coded for tracking signal changes over time from baseline to days 1, 3 and 5

The imaging agent, ^99m^Tc-duramycin, binds dead and dying cells; and the radioactivity uptake from ^99m^Tc-duramycin is positively correlated to the degree of tissue damage [15, 28, 29]. The in vivo imaging data enabled the assessment of spatiotemporal patterns in tumor response in the same individuals over the course of the study. The changes in radioactivity uptake in the tumor in response to bortezomib treatment over time is shown in Fig. 1. The ratios of mean radioactivity uptake in the tumors in bortezomib-treated over control are 1.41 (*P* = 0.022), 2.48 (*P* = 0.004) and 2.80 (*P* = 0.012) on days 1, 3 and 5 of treatment. This was compared to a ratio of 0.85 (*P* = 0.42) at baseline before bortezomib administration. The data indicated that bortezomib treatment resulted in detectable tumor response in terms of radioactivity uptake in 1 day, followed by a continued signal elevation throughout the course of the study. Individual tumor response over time with bortezomib treatment or control is shown in Fig. 1B and C, respectively.

**Fig. 2:**
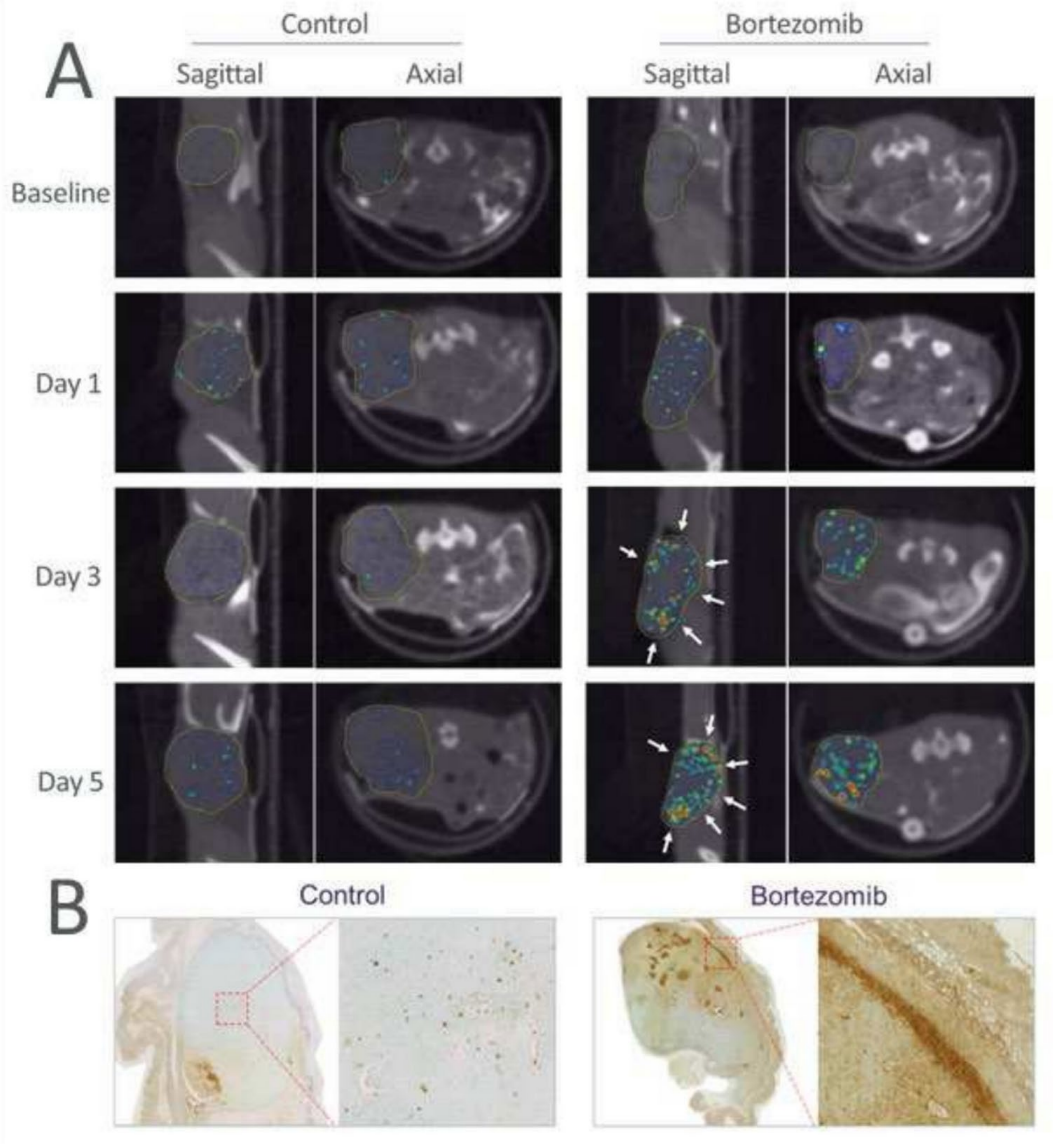
Imaging tumor response after bortezomib treatment. **A** While there was diffusive signal throughout the tumor tissue in control, more intense signal was distributed to the periphery of the tumor with Bortezomib treatment. The tumor was indicated with a yellow outline; the distribution of radioactivity uptake in the tumor periphery was marked with arrowheads. **B** TUNEL staining of excised tumor tissues on day 5, indicating diffused distribution of apoptotic nuclei throughout the tumor tissue in control. In contrast, high density of apoptotic nuclei was identified in the tumor periphery after Bortezomib treatment. The findings were consistent with in vivo imaging data

Apart from global tumoral radioactivity uptake, the in vivo imaging data provided spatial information in the tumor. As shown in Fig. 2, after bortezomib treatment, the tumor especially the periphery exhibited drastically elevated ^99m^Tc-duramycin uptake in a crescent-like pattern (Fig. 2A, arrowheads), which became visually prominent on days 3 and 5 after the start of treatment. The finding that bortezomib therapy mainly targeted peripheral regions of the tumors was validated by terminal deoxynucleotidyl transferase-mediated dUTP nick-end labeling (TUNEL) staining in treated and excised tumors, where a high density of apoptotic nuclei was seen at the rim of tumor tissue, whereas this was not the case in control tumors (Fig. 2B).

**Fig. 3:**
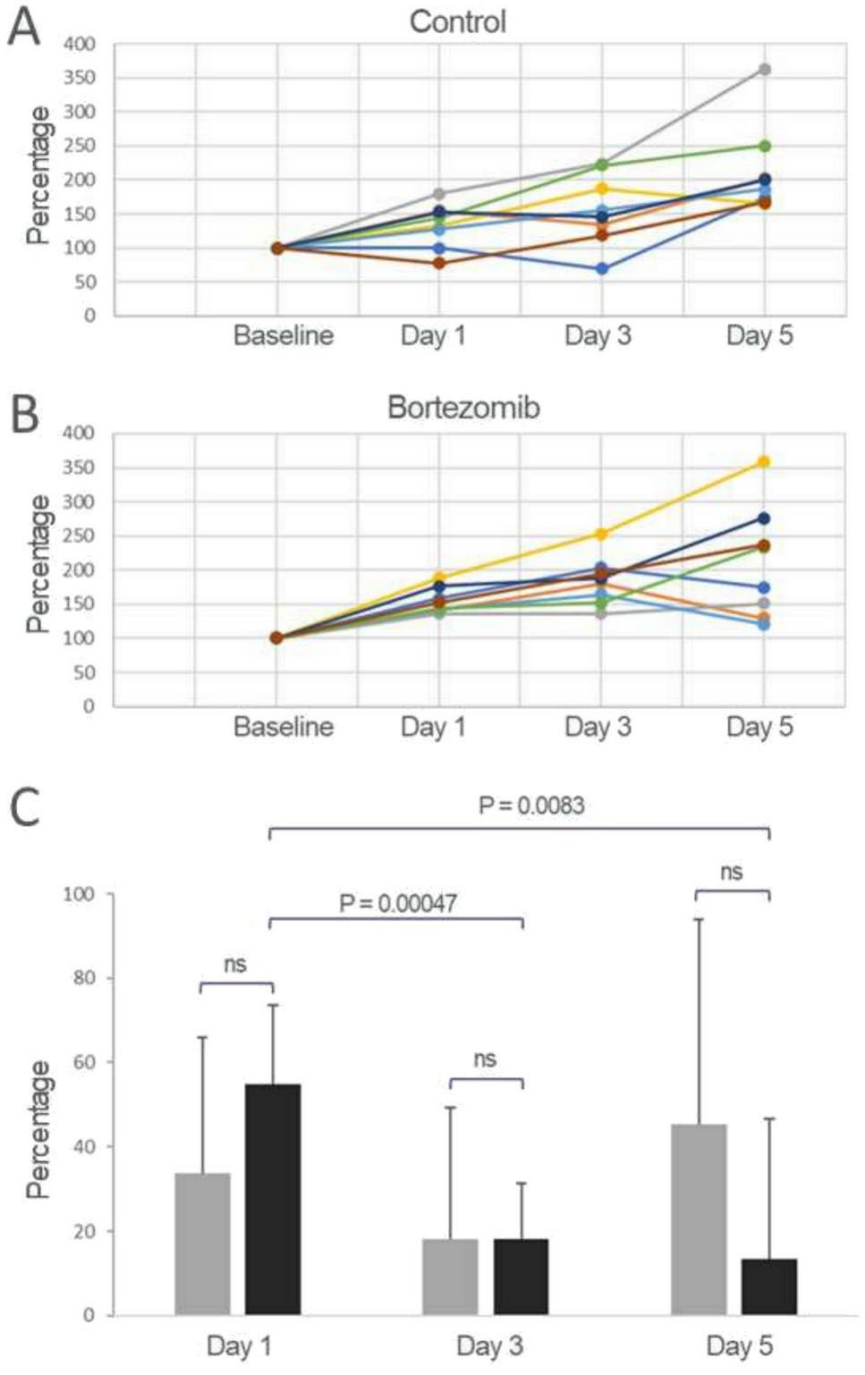
Tumor volume measurements and growth during the course of treatment. **A**, **B** Tumor volume normalized to baseline for control and bortezomib-treated groups, respectively. **C** Tumor volume change as a percentage of the previous time point (day 1 vs. baseline; day 3 vs. day 1; day 5 vs. day 3). The bortezomib-treated group exhibited a progressively lower growth rate, with a reduction in tumor growth between days 1 and 3 (*P* = 0.00047) as well as between days 1 and 5 (*P* = 0.0083)

In terms of tumor volume, data in Fig. 3 indicated that during the course of treatment, compared to control, the bortezomib-treated group exhibited a lower growth rate. On days 1, 3 and 5 post treatment, the mean tumor volume increased by 33.7, 18.0 and 45.4% for the control group. In contrast, for the bortezomib-treated group, there was a diminished tumor growth over this time, from 54.9% to 18.2%, then 13.4%. It is notable that the reduction in tumor growth between days 1 and 3 was statistically significant, with a *P* value of 0.00047, as was with day 5 (*P* = 0.0083). These changes coincided with a 2.48 (*P* = 0.004) and 2.80 (*P* = 0.012) folds increase in imaging signal on days 3 and 5, respectively, compared to control. In terms of tumor volume, there was no significant difference between control and the treated groups on any of the days.

### Assessment on systemic effects

**Fig. 4:**
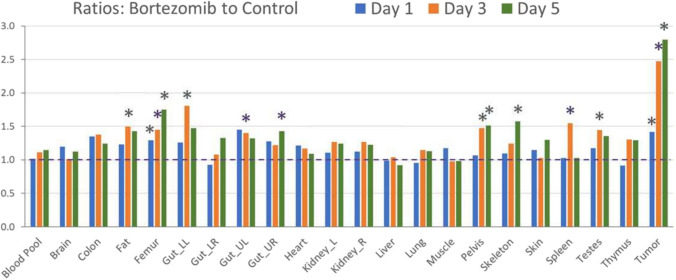
Ratios of radioactivity uptake in tissues between bortezomib and control on days 1, 3 and 5 after treatment. A ratio of 1, as shown in dotted line, indicates no change. "*" indicates tissues which had statistically significantly elevated signal compared to control

**Fig. 5:**
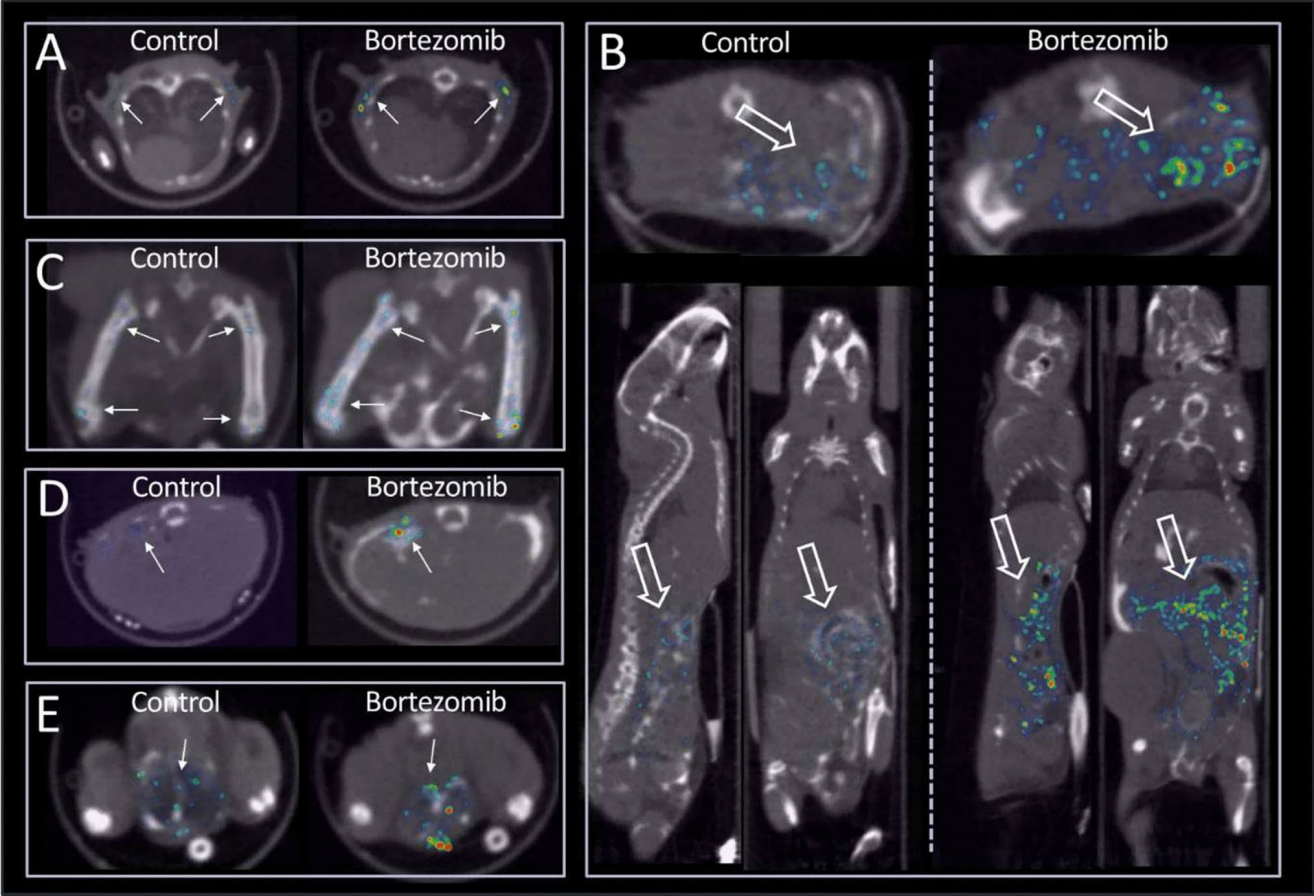
Representative images of elevated radioactivity uptake in treated tissues. **A** Adipose tissue (arrows); **B** Abdominal region (block arrows); **C** Epiphysis of the femur (arrows); **D** Spleen (arrow); and **E** Testis (arrow). Left—control, right—treated. The radioactivity uptake was normalized to the total injected radioactivity dose

**Fig. 6:**
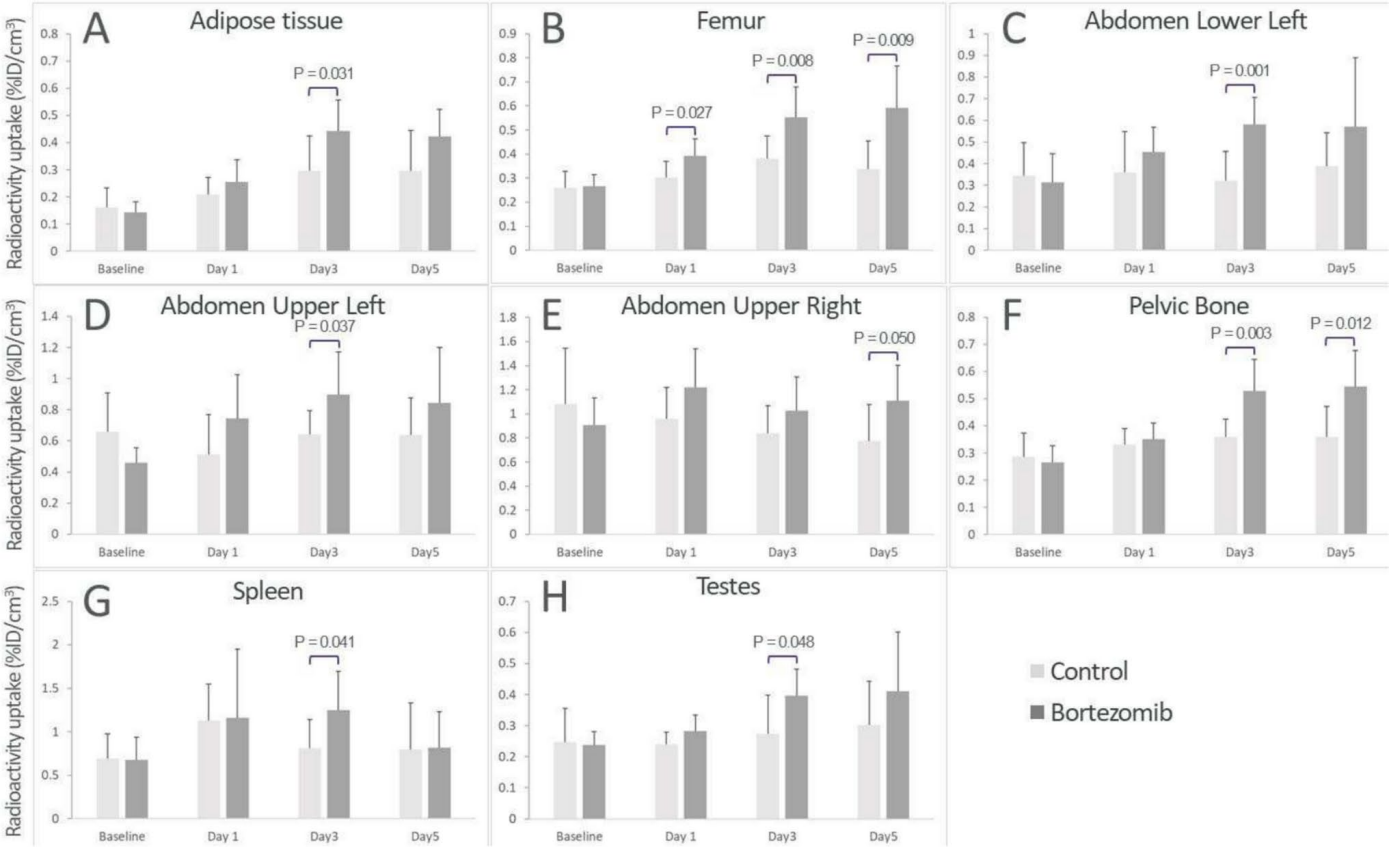
Radioactivity uptake in tissues from bortezomib treated versus control animals. The *P* values and time points at which the differences reached significance are marked for **A** Adipose tissue, **B** femur, **C**–**E** lower left, upper left and upper right quadrants of abdomen, **F** pelvic bone, **G** spleen, and **H** Testes. The radioactivity uptake was presented as the percentage of total injected radioactivity dose per cm^3^ (%ID/cm^3^)

The in vivo imaging data provided a means to semi-quantitatively identify changes as a result of drug treatment in an in vivo, whole-body approach. Overall, systemic changes after bortezomib treatment (1 mg/kg IV, Q4D) were detected in terms of the number of tissues and magnitude of signals. The degree of signal changes was shown as fold differences between treated and control; and the significance of signal changes was determined statistically based on the absolute radioactivity uptake per volume tissue, with a *P* value ≤ 0.05 considered significant. The tissues that had significant signal elevation are summarized in Table 1 with fold changes and P values from t-test and the day(s) after treatment at which these changes were detected. As shown in Fig. 4, compared to control, treatment with bortezomib resulted in detectable changes in a number of tissues. The bortezomib-treated animals experienced significant weight loss, which was accompanied with a reduction of fat volumes according to in vivo measurements. Significant radioactivity was detectable in the adipose tissue on day 3, as shown in Fig. 5A which demonstrates focal signal at the interscapular adipose tissue. To assess signal changes in the gastrointestinal tract, the abdomen was examined in 4 quadrants—upper left, upper right, lower left and lower right. Among these quadrants, the upper and lower left, as well as the upper right quadrant of the abdomen exhibited significantly elevated radioactivity uptake on days 3 or 5 after treatment (Fig. 5B) (Table 1). In the course of the treatment, there was significant signal elevation in major bones throughout the body, including the femur, pelvic bone, and the vertebrate. This also held true for the entire skeleton. Interestingly, focal signal uptake was seen in the epiphysis but not the entirety of the bone marrow (Fig. 5C). The radioactivity in the spleen was elevated on day 3 (Fig. 5D). Additionally, the imaging data identified the testes as a susceptible organ for the cytotoxic effect of bortezomib (Fig. 5E). The scatter plots for each of the positive tissues are shown in Fig. 6. Apart from these changes, the imaging results for the remaining tissues and organs did not reach statistical significance, albeit some showed elevated mean uptake signal.

**Table 1:** A summary of tissues with significant signal changes in response to bortezomib treatment

Tissue/organ	Fold change	*P* value	Time point (day)
Adipose tissue	1.49	0.031	3
Femur	1.29	0.027	1
	1.45	0.008	3
	1.75	0.005	5
Lower left quadrant of abdomen	1.81	0.001	3
Upper left quadrant of abdomen	1.40	0.037	3
Upper right quadrant of abdomen	1.43	0.050	5
Pelvic bone	1.47	0.003	3
	1.51	0.012	5
Skeleton	1.58	0.024	5
Spleen	1.54	0.041	3
Testes	1.45	0.048	3
Tumor	1.41	0.022	1
	2.48	0.004	3
	2.80	0.012	5

**Fig. 7:**
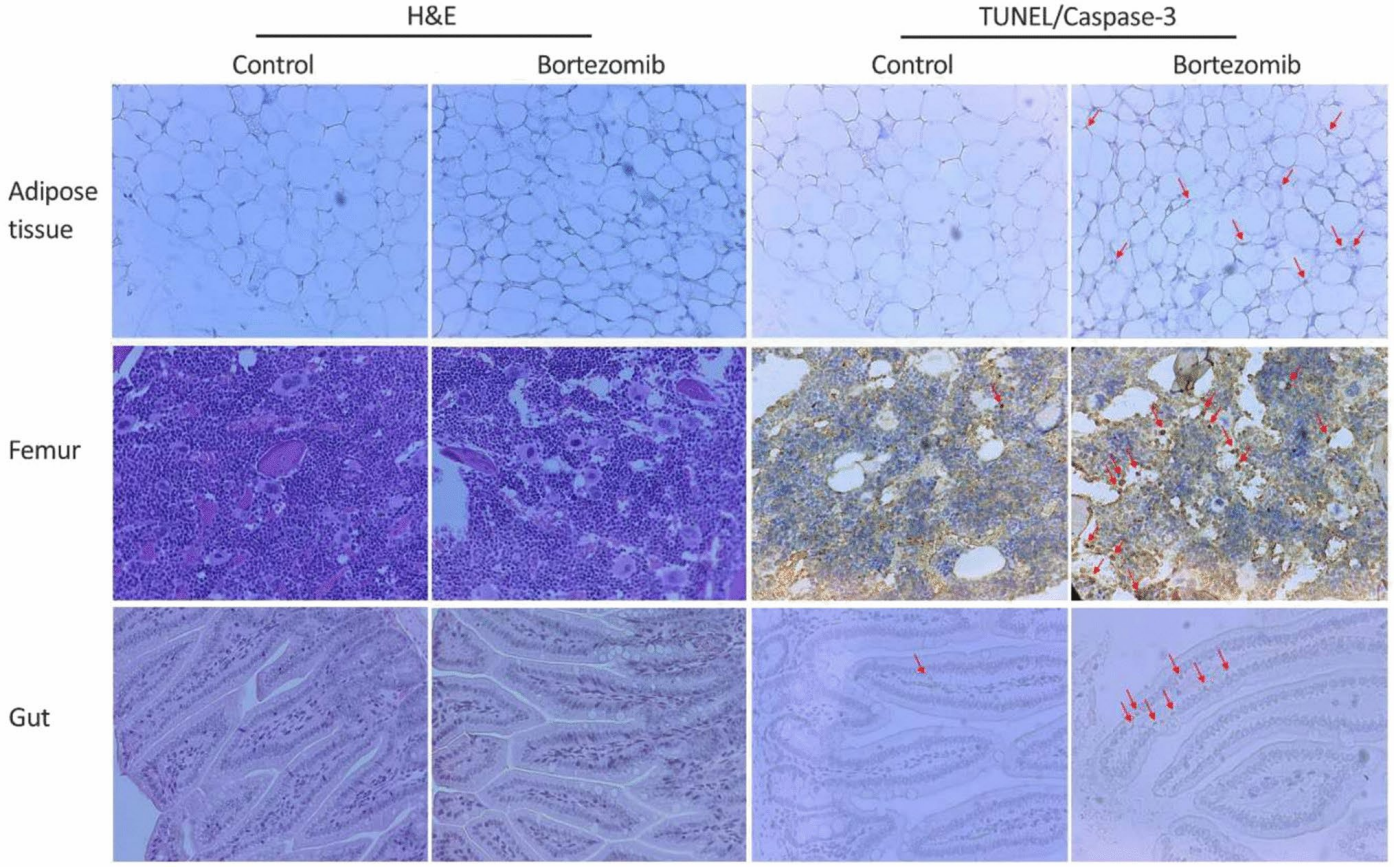
Histopathology in support of imaging data was conducted in tissues, including adipose tissue, femur and small intestine, retrieved on day-5 after treatment. Findings based on conventional H&E (left panels) were insignificant. However, elevated presence of apoptosis was detected in bortezomib-treated adipose tissue and the epiphysis of the femur using TUNEL staining (arrows), as well as the small intestine by immunochemistry for cleaved caspase-3 (arrows)

In support of the in vivo imaging studies, histopathology was conducted for control and bortezomib-treated on day-5, which according to the imaging data was a peak window where most of the changes took place. Tissues that did not exhibit significant in vivo signal changes with drug treatment in the imaging data, including the brain, heart, kidneys, liver, lung, muscle, skin and thymus were also negative by histopathology. Among susceptible tissues identified by in vivo imaging, moderate to severe level of fat necrosis was noted but no definitive finding of elevated cell death was detected based on conventional H&E staining (Fig. 7). In contrast, significantly elevated apoptotic index was determined by TUNEL staining (*P* = 0.016). Representative micrographs demonstrating scattered apoptotic nuclei in the adipose tissue are shown in Fig. 7. These findings were consistent with prominent radioactivity uptake in adipose tissues in ^99m^Tc-duramyicn SPECT. In the spleen, white pulp expansion identified in histopathology (data not shown) may be correlated with blood cell damage and elevation in splenic signal from the imaging data. ^99m^Tc-duramycin SPECT identified significantly elevated signals in the epiphysis of the femur as well as in other major bones including the pelvic bones and the vertebrate in animals. This was confirmed by the greater number of apoptotic nuclei in the epiphysis in TUNEL-stained femur sections (*P* = 0.017) (Fig. 7), whereas the findings based on H&E staining were equivocal (Fig. 7). Gastrointestinal adverse effects are known to bortezomib [26, 27]. The current in vivo imaging data identified significant signal changes in abdominal regions. Based on conventional H&E staining, no conclusive evidence of gastrointestinal toxicity was detectable (Fig. 7). However, with immunohistochemistry staining for cleaved caspase-3, regional clusters of positive staining were identified in 2 out of 3 bortezomib-treated animals, whereas this was not observed in control animals. Representative micrographs are shown in Fig. 7. Lastly, the pathological findings on the testes were nonsignificant (data not shown), while the imaging data indicated otherwise which was in agreement with prior report on oxidative-induced reproductive toxicity in male mice by bortezomib treatment [31]. However, it should be noted that the imaging data identified the time window where the signal change in the testes reached statistical significance on day-3, whereas the sampling of tissues for histopathology was timed for day-5.

Overall, based on the prevalence of signal elevation in terms of the number of tissues and magnitude of signal changes, the data were indicative of cytotoxicity-induced changes in the tumor as well as in nontumor tissues.

## Discussion

The imaging technique detects the redistribution of membrane phospholipid as a molecular marker for tissue susceptibility to cytotoxicity [15, 20, 28, 29]. Pathological cell death is a terminal cellular response and an unambiguous indicator for tissue damage. In this respect, the current technology is complementary to assays which detect changes in other aspects such as morphology, signaling, metabolism, gene and protein expression, functions and behaviors. In the current investigation, we took a whole-body imaging approach for assessing tumor and nontumor tissue response in the acute phase of bortezomib treatment. The in vivo data detected tumor response in a spatiotemporally defined fashion; at the same time, whole-body imaging identified signal changes systemically. The signal changes in space and time provided indications on the susceptibility of the tumor and nontumor tissues and the dynamic evolvement over time.

Compared to measurements on tumor size and structural changes, the current imaging technique detects the presence and distribution of tumor cell death in response to treatment. Temporally, the drastic elevation in tumor signals correlated with a reduction in tumor growth, which was consistent with a transition from proliferation to tumor cell death. Comparatively, the imaging signal in the tumors was early, indicating that the increase in imaging signal precedes changes in tumor volume or tumor growth, and is thus complementary to the anatomical measurements of the tumor. The capturing of time-dependent signal changes is valuable in outlining a time course of tumor response to treatment, which can be corroborated with the mechanism of action of a given drug. Spatially, the signal changes were located predominately along the tumoral periphery. This information is important in identifying regional susceptibility of the tumor tissue. For instance, an elevation in apoptosis in the tumor periphery is indicative of drug distribution and/or local cellular response, thus providing imaging-guided spatial information for in-depth mechanistic investigations.

Elsewhere apart from the tumor, the whole-body imaging data indicted tissue susceptibility that is consistent with damages caused by known cytotoxic side effects of the drug. Among these, significant and unambiguous signal changes in major bones throughout the body are indicative of damage in the marrow, particularly the epiphysis. These changes were in agreement with established myelosuppressive effects of bortezomib including thrombocytopenia, neutropenia and anemia [26]. Gastrointestinal impact is another major adverse effect of bortezomib [27]. The imaging data identified significant signal changes in abdominal regions. The gut is an elongated and tortuous organ, which makes it challenging to analyze in its entirety by histopathology. This is complemented by in vivo imaging, which provides volumetric data on a tissue level. This approach, in complementary with conventional histology-based methods, can help minimize sampling bias when large volumes of tissues are examined. Peripheral neuropathy is another common adverse effect in bortezomib treatment [24, 30]. However, this condition predominately involves distal axon degeneration and anomalies in mitochondria and ER, where these intracellular changes were not manifested as plasma membrane reorganization thus were not detectable to the current approach.

Outside the most common side effects, the imaging data identified susceptibility in the testes, which is consistent with bortezomib being a potent gonadal toxic drug, causing testicular toxicity by the upregulation of oxidative stress with long-term male infertility [31]. Furthermore, extensive degeneration of adipose tissue was detected with bortezomib treatment. It is known that higher body fat composition is correlated with poor treatment response [32]. Whether this is related to the elevated signals in the adipose signal remains to be investigated.

In the current study, discrepancies were identified between in vivo imaging and conventional H&E-based histopathology. As a molecular marker for cytotoxicity, the current approach detects changes in the phospholipid distribution in cellular membranes, which precede morphological changes such as cell shrinkage, nuclear fragmentation and the formation of apoptotic bodies– markers that are recognized in histopathology [33]. Additionally, the imaging data identify the overall signal changes volumetrically on a macroscopic scale, which complements histopathology’s microscopic nature. These technological differences between the two methods may explain why certain tissues had signal changes detectable by in vivo imaging but not conventional H&E-based histopathology, and that part of the discrepancy was addressed by additional TUNEL staining. This outcome suggests that the imaging approach may be more sensitive for detecting cytotoxicity-induced tissue damage, and the imaging agent may enable the detection of changes that are otherwise inconspicuous to conventional histopathology. Another factor that may have contributed to the discrepancy between imaging and histopathology was the timing of sampling. While the former, due to its minimally invasive nature, is applicable repeatedly in a longitudinal fashion, the latter is terminal in order to retrieve tissues for analyses. These features warrant future in-depth studies for comparing the sensitivity and specificity between the two methods.

The in vivo imaging data, based on radioactivity uptake from a targeted imaging agent, are a semi-quantitative manifestation of underlying tissue response to cytotoxicity. The data can potentially allow for magnitude-based comparison on a tissue or a set of tissues between treatments. When put into perspective, the current approach can be useful in ranking drug candidates in terms of cytotoxic impact and off-target tissue susceptibility within a given tissue versus antitumor potency/efficacy, and thus potentially help identify safer and more efficacious pharmaceuticals for prioritization.

Finally, the minimally invasive nature of the imaging approach allows the acquisition of in vivo data at multiple time points from the same cohorts of animals over time. This practice enables dynamic data from each individual for capturing the spatiotemporal evolvement of signal changes. Data can be extracted on an individual basis, thus with personalized information in a study setting that will otherwise results in heterogeneous response across different individuals.

Overall, the current approach provides useful and complementary information in multiple aspects. The imaging data are whole-body and volumetric, with measurements on regional, tissue, and organismal levels. The tissue uptake of ^99m^Tc-duramycin, which detects phospholipid reorganization, leads to semi-quantitative and global measurements. With this information and relatively fast turnaround time, the current technology is potentially useful in drug development and optimization of therapies by identifying efficacious and safe drugs for translation.

## Materials and methods

### Multiple myeloma xenograft model

The animal protocol was approved by the Institutional Animal Care and Use Committee under the National Institutes of Health guideline. OPM-2 multiple myeloma cells were purchased from the DSMZ-German Collection of Microorganisms and Cell Cultures HmbH. Xenograft OPM-2 multiple myeloma tumors were grown at the lower left frank of male SCID-beige mice (8–10 weeks old) by inoculating approximately 5 million cells subcutaneously in 0.2 ml of MEM and Matrigel (1:1 vol:vol).

### Chemotherapeutic dosing preparation

Bortezomib (Sigma-Aldrich, St. Louis, MO) was dissolved in a small volume of DMSO as a stock solution. At the time of administration, the solution was diluted in saline (1:20 v/v) and sterile filtered.

### Dosing of animals

When the tumor reached approximately 5 mm in diameter, baseline scan was conducted (see below for details), then the animals were dosed with bortezomib at 1 mg/kg, once every 4 days, intravenously.

### Imaging

Two days after the baseline scan, drug treatment was started according to study group. Two groups were enrolled at 8 tumor-bearing mice each, including control and bortezomib. Four hours after drug administration, ^99m^Tc-duramycin SPECT/CT was acquired as described below. Scans were repeated on days 1, 3 and 5. ^99m^Tc-duramycin was prepared as previously described [15]. Each mouse was anesthetized with isoflurane and ^99m^Tc-duramycin was injected intravenously at a dose of approximately 0.2 to 0.3 mCi via the tail vein. SPECT imaging data were acquired at an hour later on a U-SPECT + /CT (MILabs, Netherlands) using a general-purpose mouse collimator with 1.0 mm pinholes and 0.6 mm resolution. Two 20 min sets of data (frames) were acquired and combined. Pixel-based ordered subset expectation maximization (POSEM) reconstruction was used with 4 subsets, 6 iterations, and a 3D-Gaussian kernel (FWHM of 0.8 mm) filter. X-ray microCT was used for anatomic guidance and attenuation correction. The collimator was calibrated with a ^99m^Tc point source to enable conversion from count per second (cps) to MBq. Imaging data were checked for completeness by the operators at the end of acquisition and reconstructed using inbuilt automated functions on the scanner computer.

### Imaging data analysis

The reconstructed image files were imported to and analyzed with Amira software (Life Science Research—Thermo Fisher). Co-registration and segmentation were completed as described previously [15]. The individual segmentation result contained fitted geometries with known volumes for each region of interest (ROI). The SPECT signal was determined in terms of radioactivity decay per second, and converted to percentage of injected dose per cm^3^ (%ID/cm^3^). Of the enrolled tumor-bearing mice per group, 8 data sets were found useable for control at all time points, 8 data sets were useable for bortezomib except for 1 mouse on day 5. Tumor volume was determined based on CT. For radioactivity uptake data analysis, mixed-effects models were used to examine the changes in outcomes of interest (ie: in different tissues) from baseline (Day 0) to each follow-up day (Day 1, 3, 5) among groups taking into account variability within and across mice. The groups and time points were modeled as fixed effects; and the mice were modeled as the random effect. A *P* value of ≤ 0.05 is considered statistically significant.

### Histology

A parallel group of three tumor-bearing animals, including control and treatment with bortezomib, were euthanized on day-5, where the signal changes were at or near peak intensities according to the in vivo imaging data. The tumors and other tissues were excised and fixed in 10% formalin and 4-micron paraffin sections were cut. H&E sections were examined by a certified clinical pathologist in a blinded fashion. The pathology findings were scored from 0 to 3, where 0 is no abnormality, 1 being low, 2 being moderate and 3 being severe. Additionally, slides were stained using the TUNEL assay for the detection of apoptotic nuclei in the sections. Alternatively, the presence of cleaved caspase-3 was assessed using immunohistochemistry with a primary antibody against activated caspase-3.

## Data Availability

No datasets were generated or analysed during the current study.
